# PLP2 Expression as a Prognostic and Therapeutic Indicator in High-Risk Multiple Myeloma

**DOI:** 10.1155/2020/4286101

**Published:** 2020-06-10

**Authors:** Hua Bai, Yudi Zhu, Peipei Xu, Bing Chen

**Affiliations:** Department of Hematology, Nanjing Drum Tower Hospital Clinical College of Nanjing Medical University, Nanjing 210008, China

## Abstract

Multiple myeloma (MM) is a devastating cancer with a highly heterogeneous outcome. Because of the heterogeneity of myeloma cells, risk stratification is important for making therapeutic regimens. Nevertheless, no immunohistochemical predictive and prognostic marker has been constructed yet. In the present study, we explored the prognostic value of proteolipid protein 2 (PLP2) in MM patients using immunohistochemistry (IHC). We assessed PLP2 expression in bone marrow (BM) biopsy specimens obtained from 87 newly diagnosed MM (NDMM) patients. Correlations between PLP2 expression and clinicopathological features were analyzed. PLP2 expression was present in high-risk MM patients, which was increased with disease progression and poor prognosis. PLP2 was increasing in parallel with high beta-2 microglobulin (*β*2-MG) and lactate dehydrogenase (LDH). Furthermore, MM patients with low PLP2 expression could achieve a favorable treatment response. PLP2 may be a novel biomarker for prognostic prediction and a therapeutic target for anti-MM treatments.

## 1. Introduction

Multiple myeloma (MM) is an incurable plasma cell disease and is characterized by hypercalcaemia, anaemia, lytic bone lesions, and renal disorder. Although chemotherapies have obviously conferred survival advantage, MM remains to be a relapsed or refractory disease [1–3]. Thus, continued investigations to identify biomarkers for risk stratification are still an urgent requirement [4]. MM is characterized by pathological and clinical heterogeneity, and eight molecular subgroups have been established [5, 6], which contribute to the heterogeneous outcomes of MM. Despite these developments and various parameters such as serum ALB, *β*2-MG and monoclonal proteins being widely adopted as a standard staging system (DS/ISS), they are still inadequate in making therapeutic decisions [7]. The independent biomarkers that can categorize MM patients according to laboratory parameters and clinical treatment responses are still lacking. Consequently, the discrimination of high-risk MM patients using adequate biomarkers at the initial period is important to reduce relapse and obtain durable remission [8, 9].

Proteolipid protein 2 (PLP2) is an integral ion channel membrane protein of the endoplasmic reticulum (ER) [10]. While the exact function of PLP2 under normal conditions is not known, the study of the protein has revealed several features in ER. Firstly, it is an integral membrane protein that localizes to the ER. Secondly, PLP2-knockout mice display increased ER stress in neurons under hypoxia, which leads to apoptotic cell death [11]; downregulation of PLP2 increased ER stress-induced apoptosis and reduced tumor cell survival in vitro [10]. In contrast, proteasome inhibition (bortezomib) induces ER stress due to accumulation of misfolded and unfolded proteins in the ER, which leads to myeloma cell death [12]. Based on these findings, we hypothesized that PLP2 may contribute to eradication of MM cells that escape bortezomib-induced apoptosis, potentially improving MM cell survival. Recently, this protein was reported to be involved in several human cancers [13–16]. Sonoda et al. demonstrated that PLP2 enhances cell proliferation, adhesion, and invasion in melanoma [15]; Xiao et al. showed that PLP2 is significantly upregulated and predicts poor prognosis in renal cell carcinoma patients [17]; and Feng et al. found that high PLP2 expression predicts an aggressive disease grade and a shorter survival in glioma patients [10]. However, the potential roles of PLP2 in MM remain elusive.

In the present study, we explored the prognostic value of PLP2 expression in MM patients using immunohistochemistry (IHC) and Gene Expression Omnibus (GEO) datasets. We found that PLP2 expression correlates with tumor progression and poor prognosis in MM. And our investigation verified that PLP2 could be an efficient predictor of clinical outcome at gene and protein levels.

## 2. Materials and Methods

### 2.1. Microarray Analysis

GEO datasets were adopted to measure the gene expression of *PLP2* in MM patients (GSE5900 [18], GSE2658 [5], GSE24080 [19], and GSE9782 [20]). Data acquisition and normalization methods in the above databases have been described previously [19]. The gene expression of *PLP2* in myeloma cells was determined using the Affymetrix U133 Plus 2.0 microarray, which was performed as previously described [5, 21].

### 2.2. Patients and Clinical Features

This study analyzed the PLP2 expression in BM biopsy specimens collected from 87 NDMM patients from January 2013 to December 2019 at Nanjing Drum Tower Hospital Clinical College of Nanjing Medical University; diagnoses were in accordance with the 2008 World Health Organization criteria, and the curative effect standards were approved by the International Myeloma Working Group (IMWG). The clinical features were procured from medical records, including age, sex, and serological markers; the details of MM patients' characteristic are shown in Table 1. The primary induction therapies for these NDMM patients were bortezomib-based regimens.

### 2.3. IHC and Pathologic Evaluation

Morphological findings were obtained using H&E stains to confirm an appropriate amount of tumor cells. Formalin-fixed, paraffin-embedded sections were utilized for IHC with the following antibodies: CD138 (Proteintech, USA) and PLP2 (Proteintech, USA), according to the manufacturer's protocol [22]. Aggregates of plasma cells were assessed for CD138 and PLP2 in sequential slides [23]. Firstly, each slide was observed to choose areas with aggregates of plasma cells. Then, these slides detected the staining intensity of PLP2. Without prior knowledge of patients' outcome, two pathologists independently graded the immunostaining intensity as follows: 0, <10% of tumor cells or no staining; 1+, ≥10% tumor cells with weak staining intensity; 2+, ≥10% tumor cells with moderate staining intensity; and 3+, ≥10% tumor cells with strong staining intensity.

### 2.4. Statistical Analysis

Various statistical analyses were utilized to evaluate the roles of PLP2 expression in clinicopathological features and prognosis in MM patients. Two-tailed Student's *t*-test and one-way analysis of variance were adopted to compare two or multiple experimental groups. The chi-square test was utilized to compare clinicopathological features between the high/low PLP2 expression subgroups. Survival curves were plotted according to the Kaplan-Meier method, and the log-rank test was employed to analyze significance between survival curves. The effect of PLP2 expression on outcome was analyzed using univariate and multivariate Cox regression models. For our analyses, the GraphPad Prism 6 software was employed and ^∗^*p* ≤ 0.05 was considered statistically significant.

## 3. Results

### 3.1. PLP2 Was a High-Risk Myeloma Gene

**Figure 1:**
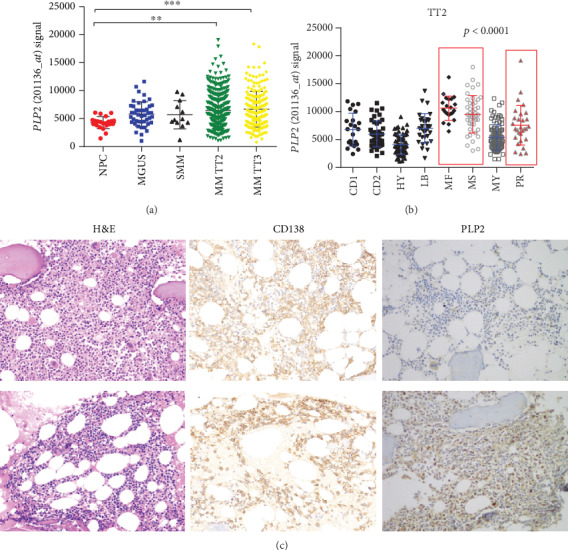
*PLP2* was a high-risk myeloma gene. (a) *PLP2* expression of NPC (*n* = 22), MGUS (*n* = 44), SMM (*n* = 12), and MM (TT2, *n* = 351; TT3, *n* = 208) in GSE5900 and GSE2658 datasets (^∗∗^*p* < 0.01, ^∗∗∗^*p* < 0.001). (b) A scatter plot showing the *PLP2* expression in eight MM subgroups (CD1 and CD2 subgroups with spiked expression of CCND1 and CCND3; PR: proliferation; LB: low-bone disease; HY: hyperdiploid; MS: MMSET; MF: MAFB; MY: myeloid). (c) CD138 and PLP2 expressions in the bone marrow of NDMM patients. Representative case with a lack of PLP2 expression: H&E stain, CD138 immunostain, and PLP2 immunostain (upper row). Representative case with stable PLP2 expression: H&E stain, CD138 immunostain, and PLP2 immunostain (lower row).

To assess the potential that PLP2 is crucial for myeloma, we examined *PLP2* expression in the normal plasma cell (NPC), monoclonal gammopathy of undetermined significance (MGUS), smoldering multiple myeloma (SMM), and MM patients using GEO datasets. Notably, *PLP2* expression increased significantly from NPC, SMM, MGUS to MM TT2 (Total Therapy 2) and TT3 samples (^∗∗^*p* < 0.001, Figure 1(a)). In detail, we investigated whether heightened *PLP2* expression in the MM TT2 cohort might be related to particular molecular subgroups.  Figure 1(b) presents the *PLP2* expression in 8 molecular subgroups, showing that elevated *PLP2* expression was prevalent in 3 known to confer high risk in terms of clinical outcome and course: proliferation (PR), MAF/MAFB (MF), and MMSET/FGGR3 (MS) (*p* < 0.001). These findings prompted us to confirm that *PLP2* is a high-risk gene in MM.

### 3.2. Correlations between PLP2 Expression and Clinicopathological Features

**Figure 2:**
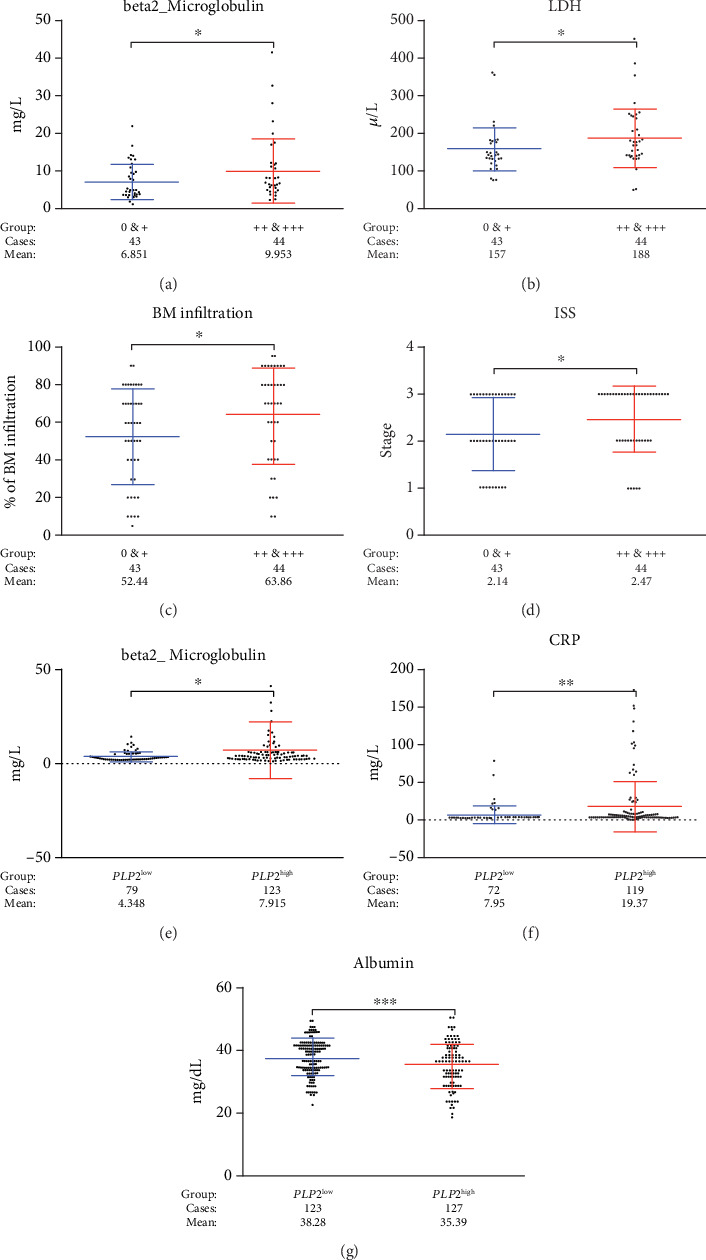
PLP2 was linked to myeloma progression. (a–d) *β*2-MG, LDH, bone marrow infiltration, and ISS stages were expressed the highest in the high PLP2 expression subgroup, while the lowest in the low PLP2 expression subgroup (^∗^*p* < 0.05). (e–g) *β*2-MG and CRP were expressed the highest in the *PLP2*^high^ subgroup, while ALB was expressed the lowest in the *PLP2*^high^ subgroup (^∗∗^*p* < 0.01, ^∗∗∗^*p* < 0.001).

To evaluate PLP2 expression in MM bone marrow, we performed IHC for PLP2 and divided 87 cases into two subgroups according to the immunostaining intensity (Figure 1(c)). Forty-four patients (50%) were classified into the high PLP2 expression subgroup, depending on the cut-off (2+). The clinicopathological features according to the PLP2 expression are listed in Table 1. No significant correlations were detected between PLP2 and other clinicopathological features, such as sex, age, serum creatinine (sCr), haemoglobin (HB), and erythrocyte sedimentation (ESR). Strong PLP2 staining intensity was significantly associated with high *β*2-MG, LDH, BM infiltration levels, and ISS stages (^∗^*p* < 0.05, Figures 2(a)–2(d)). Consistent with our finding, high *PLP2* gene expression was also significantly correlated with high *β*2-MG, C-reactive protein (CRP), and low ALB levels in GSE9782 (^∗^*p* < 0.05, Figures 2(e)–2(g)).

### 3.3. Increased PLP2 Expression Correlated with Poor Prognosis in MM

**Figure 3:**
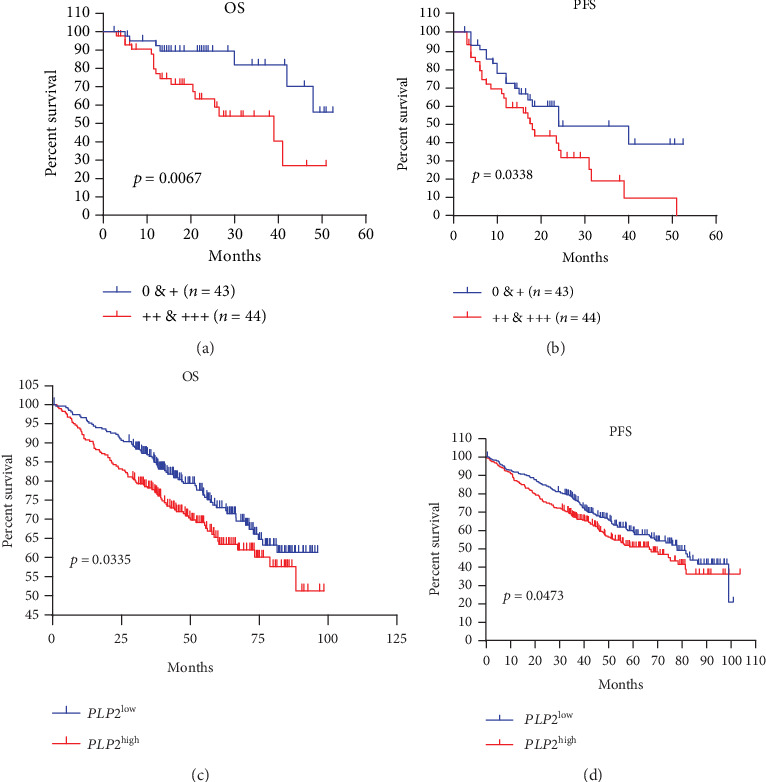
High PLP2 expression was linked to a poor prognosis in two independent datasets. (a, b) Kaplan-Meier analyses of OS and PFS revealed that strong PLP2 staining intensity conferred inferior clinical outcomes in our cohort. (c, d) Kaplan-Meier analyses of OS and PFS revealed that high *PLP2* gene expression conferred inferior clinical outcomes in GSE24080.

To investigate the correlation of survival time and PLP2 expression in MM, we performed the Kaplan-Meier survival analysis in two groups. The high PLP2 expression subgroup (2+ and 3+) had shorter median overall survival (OS) and progression-free survival (PFS) time than the low PLP2 expression subgroup (0 and 1+) (OS: 15.5 vs. 21.5 months; PFS: 12 vs. 15 months). As shown in Figures 3(a) and 3(b), MM patients with strong PLP2 staining intensity had an inferior OS (*p* = 0.0067) and PFS (*p* = 0.0338). In addition, to evaluate PLP2 expression and clinicopathological features on outcomes, we utilized the univariate and multivariate Cox analyses. Based on the results of the univariate Cox proportional hazard regression analysis, LDH, *β*2-MG, and PLP2 expression (OS: HR = 3.250, 95% CI: 1.320-7.999, *p* = 0.010, Table 2; PFS: HR = 1.865, 95% CI: 1.031-3.375, *p* = 0.039, Table 3) were included in the multivariable Cox proportional hazard regression analysis, which indicated that the PLP2 expression was still an independent prognostic factor in terms of OS in 87 MM patients (HR = 2.598, 95% CI: 1.032-5.991, *p* = 0.041, Table 2). We also applied the Kaplan-Meier analysis to validated *PLP2* gene expression in another independent dataset, and the Kaplan-Meier survival analysis suggested that patients in the *PLP2*^low^ subgroup had better OS and PFS compared with those in the *PLP2*^high^ subgroup in GSE24080 (*p* = 0.0335, Figure 3(c); *p* = 0.0473, Figure 3(d)).

### 3.4. Treatment Response to Bortezomib-Based Regimens

In addition, compared to the high PLP2 expression subgroup (2+ and 3+), patients with low PLP2 expression significantly responded to bortezomib-based regimens evidenced by the increased objective response rate (ORR, *p* = 0.0128), ≥very good partial remission (VGPR, *p* = 0.0189), ≥complete remission (CR, *p* = 0.0127), and ≥stringent CR (sCR, *p* = 0.0091) (Table 4). These data strongly suggested that PLP2 expressions are linked to treatment response to bortezomib-based regimens.

## 4. Discussion

MM remains incurable despite novel treatments, and plenty of prognostic biomarkers that reflect host- or tumor-related factors have failed to explain thoroughly the heterogeneity in clinical outcome and course [24]. To stratify risk stratification for MM patients, some evaluation systems had been established using prognostic parameters [25, 26]. Thus, evaluating clinical markers of MM is crucial for predicting the prognosis and making personalized treatment regimens. In the present study, *PLP2* was expressed significantly higher in aggressive subgroups (MS, MF, and PR), which were characterized by high-risk myeloma and related to an adverse prognosis [5, 27].

Recently, PLP2 has been reported to be associated with tumor aggressiveness and poor prognosis. Sonoda et al. showed that upregulation of PLP2 plays vital roles in activation of the PI3K/AKT signaling and promotion of melanoma cell proliferation [15]. In addition, the downregulation of PLP2 by microRNA-664 obviously inhibited melanoma and leukemic cell invasion and proliferation [16, 28]. But to our knowledge, our study was the first report indicating a relationship between PLP2 expression and prognosis in MM patients. As PLP2 expression could be quickly evaluated by IHC, immunostaining intensity of PLP2 would be an advantageous biomarker for identifying high-risk MM with poor prognosis.

In this study, we analyzed the prognostic significance of PLP2 expression in MM patients using IHC analysis and GEO datasets and correlated with markers of myeloma activity, such as lower serum levels of ALB, higher serum levels of *β*2-MG, LDH, and CRP. Among them, ISS has been constructed which combined serum markers of tumor burden (ALB and *β*2-MG) with markers of aggressive tumor biology (LDH) [7, 29]. Whether the ISS is used or not, ALB and renal function have been considered easy and good indicators of survival [30]. The serum level of *β*2-MG is one of the most important independent predictors of survival and considered an indicator of tumor burden [31]. High levels of circulating CRP enhanced MM cell proliferation and drug resistance under stressed conditions [32, 33]. More importantly, in our cohort, PLP2 expression correlated significantly to all the aforementioned parameters of disease activity, whereas these correlations still remained for ALB, *β*2-MG, and CRP in GSE9782. Our results supported the fact that PLP2 expression has prognostic values. The high PLP2 expression subgroup had a significantly shorter OS and PFS in our cohort, whereas analysis of GSE24080 was also consistent with our finding. Furthermore, multivariate Cox analysis indicated that MM patients with high PLP2 expression have poor prognosis independently from other clinical features, although these clinical features are the powerful prognostic indicators in MM patients [33]. Consequently, PLP2 can facilitate tumor burden and influence the prognostic impact on MM.

Another important finding was that the PLP2 expression appears to correlate in response to bortezomib-based chemotherapy. Bortezomib, which targets the 26 s proteasome subunit *β*5, has induced high percentage of response rates [34, 35]. However, resistance to bortezomib in MM is the major concern, prompting the development of novel targeted therapy. An oblivious variance was exhibited in our cohort; MM patients with low PLP2 expression could achieve a favorable treatment response (sCR, CR, VGPR, and PR). This also highlights the probability that the decreased PLP2 expression could be of interest as a new predictive marker of favorable treatment responses and indicates new potential mechanisms of the therapeutic molecules.

## 5. Conclusions

In summary, PLP2 was a bone fide high-risk MM marker that correlated with a poor outcome in newly diagnosed MM patients. Incorporation of PLP2 expression into risk determination algorithms for MM patients will facilitate the development of bortezomib-based treatments.

## Figures and Tables

**Table 1:** Relation of the characteristics in 87 NDMM patients.

Characteristic	No. of patient/total no. (%)	0 & + (*n* = 43)	++ & +++ (*n* = 44)	*p* value
Age ≥ 65 yr	28/87 (32)	16/43 (37)	12/44 (27)	0.321^†^
Male sex	44/87 (50)	26/43 (60)	18/44 (40)	0.068^†^
*β*2‐MG ≥ 3.5 mg/L	70/87 (80)	29/43 (67)	41/44 (93)	0.002^†^
sCr ≥ 176.8 *μ*mol/L	19/87 (21)	13/43 (30)	6/44 (13)	0.073^∗^
LDH ≥ 170 U/L	30/87 (34)	8/43 (18)	22/44 (50)	0.002^†^
CRP ≥ 4 mg/L	27/87 (31)	15/43 (34)	12/44 (27)	0.492^∗^
ESR ≥ 100 mm/H	40/87 (45)	18/43 (41)	22/44 (50)	0.521^∗^
HB ≥ 100 g/L	34/87 (39)	17/43 (39)	17/44 (38)	0.931^†^
ALB ≥ 35 g/L	35/87 (40)	16/43 (37)	19/44 (43)	0.663^∗^

Abbreviations: sCr: serum creatinine; CRP: C-reactive protein; ESR: erythrocyte sedimentation; ALB: serum albumin; *β*2-MG: *β*2-microglobulin; LDH: lactate dehydrogenase. ^∗^Fisher's exact test was used. ^†^The chi-square test was used.

**Table 2:** Univariate and multivariate Cox regression analyses for OS in 87 NDMM patients.

Variables	Univariate model			Multivariate model		
	HR	95% CI	*p*	HR	95% CI	*p*
Age ≥ 65 yr	1.072	0.467-2.460	0.870			
Male sex	0.290	0.035-2.431	0.254			
*β*2 − MG ≥ 3.5 mg/L	1.669	1.268-2.182	0.032	1.349	0.976-3.168	0.171
SCr ≥ 176.8 *μ*mol/L	0.567	0.192-1.672	0.304			
LDH ≥ 170 U/L	3.561	1.643-6.154	0.015	2.592	1.129-5.611	0.010
CRP ≥ 4 mg/L	1.415	0.534-3.754	0.485			
ESR ≥ 100 mm/H	1.517	0.622-3.699	0.359			
HB ≥ 100 g/L	0.573	0.226-1.455	0.241			
ALB ≥ 35 g/L	1.501	0.631-3.435	0.336			
PLP2 ++ & +++	3.250	1.320-7.999	0.010	2.598	1.032-5.991	0.041

**Table 3:** Univariate and multivariate Cox regression analyses for PFS in 87 NDMM patients.

Variables	Univariate model			Multivariate model		
	HR	95% CI	*p*	HR	95% CI	*p*
Age ≥ 65 yr	1.316	0.726-2.384	0.366			
Male sex	1.051	0.138-7.987	0.962			
*β*2 − MG ≥ 3.5 mg/L	1.934	1.291-6.798	0.023	1.679	0.647-3.965	0.171
SCr ≥ 176.8 *μ*mol/L	1.102	0.569-2.133	0.774			
LDH ≥ 170 U/L	1.645	1.525-4.087	0.067
CRP ≥ 4 mg/L	1.194	0.586-2.432	0.625			
ESR ≥ 100 mm/H	1.070	0.545-2.101	0.845			
HB ≥ 100 g/L	0.773	0.421-1.423	0.409			
ALB ≥ 35 g/L	1.757	0.973-3.171	0.062			
PLP2 ++ & +++	1.865	1.031-3.375	0.039	1.549	0.988-2.386	0.095

**Table 4:** PLP2 was related to treatment response. The correlations between drug responses with PLP2 levels were analyzed by chi-square test. Patients with low PLP2 expression significantly responded to chemotherapies evidenced by increased ORR, ≥VGPR, ≥CR, and ≥sCR.

	No. of patients/total no. (%)	0 & + (*n* = 43)	++ & +++ (*n* = 44)	*p* value
ORR	67/87 (77)	38/43 (88)	29/44 (65)	0.0128
≥VGPR	54/87 (62)	32/43 (74)	22/44 (50)	0.0189
≥CR	35/87 (40)	23/43 (53)	12/44 (27)	0.0127
≥sCR	19/87 (21)	15/43 (34)	5/44 (11)	0.0091

## Data Availability

The data used to support the findings of this study are available from the corresponding author upon request.
